# Texture Analysis of T2-Weighted MR Images to Assess Acute Inflammation in Brain MS Lesions

**DOI:** 10.1371/journal.pone.0145497

**Published:** 2015-12-22

**Authors:** Nicolas Michoux, Alain Guillet, Denis Rommel, Giosué Mazzamuto, Christian Sindic, Thierry Duprez

**Affiliations:** 1 IREC (Institute of Experimental and Clinical Research), Université Catholique de Louvain, Brussels, Belgium; 2 Department of Statistics, Université Catholique de Louvain, Brussels, Belgium; 3 Department of Radiology, Cliniques Universitaires Saint-Luc, Université Catholique de Louvain, Brussels, Belgium; 4 Department of Neurology, Cliniques Universitaires Saint-Luc, Université Catholique de Louvain, Brussels, Belgium; University of Minnesota, UNITED STATES

## Abstract

Brain blood barrier breakdown as assessed by contrast-enhanced (CE) T1-weighted MR imaging is currently the standard radiological marker of inflammatory activity in multiple sclerosis (MS) patients. Our objective was to evaluate the performance of an alternative model assessing the inflammatory activity of MS lesions by texture analysis of T2-weighted MR images. Twenty-one patients with definite MS were examined on the same 3.0T MR system by T2-weighted, FLAIR, diffusion-weighted and CE-T1 sequences. Lesions and mirrored contralateral areas within the normal appearing white matter (NAWM) were characterized by texture parameters computed from the gray level co-occurrence and run length matrices, and by the apparent diffusion coefficient (ADC). Statistical differences between MS lesions and NAWM were analyzed. ROC analysis and leave-one-out cross-validation were performed to evaluate the performance of individual parameters, and multi-parametric models using linear discriminant analysis (LDA), partial least squares (PLS) and logistic regression (LR) in the identification of CE lesions. ADC and all but one texture parameter were significantly different within white matter lesions compared to within NAWM (*p* < 0.0167). Using LDA, an 8-texture parameter model identified CE lesions with a sensitivity Se = 70% and a specificity Sp = 76%. Using LR, a 10-texture parameter model performed better with Se = 86% / Sp = 84%. Using PLS, a 6-texture parameter model achieved the highest accuracy with Se = 88% / Sp = 81%. Texture parameter from T2-weighted images can assess brain inflammatory activity with sufficient accuracy to be considered as a potential alternative to enhancement on CE T1-weighted images.

## Introduction

Multiple Sclerosis (MS) is a chronic autoimmune inflammatory disease of the central nervous system featured by the onset of multifocal white matter (WM) inflammatory foci resulting in irreversible parenchymal damage. Shortly after its introduction in clinical practice, magnetic resonance imaging (MRI) became the most sensitive imaging modality in the detection of chronic lesions as well as the assessment of inflammatory activity [1].

Conventional MR examination usually includes fluid attenuated inversion recovery (FLAIR) and T2-weighted (T2-W) imaging for lesion load delineation, together with contrast-enhanced (CE) T1-weighted (T1-W) imaging to detect foci of brain blood barrier (BBB) disruption due to local inflammation. Diffusion-weighted imaging (DWI), from which mapping of the apparent diffusion coefficient (ADC) is derived, may give additional information about cell loss and/or ultrastructural disorganization within diseased parenchyma. Though diffuse involvement of the CNS with the MS disease process has been highlighted by histopathological studies, acute inflammatory foci occur, which may be assessed either by the *a posteriori* demonstration of lesion size enlargement and/or *de novo* lesion appearance on serial T2-W images at the chronic phase, or by contemporary contrast-enhancement on T1-W images of a single examination at acute phase [2]. In the latter condition, the BBB breakdown allows leakage of the gadolinium chelates from the vascular compartment to intercellular interstitium resulting in a local T1 time shortening of adjacent spins producing hyper signal intensity on CE T1-W images. Despite recent technical advances in DW and diffusion tensor imaging, changes in diffusion parameters in MS remain equivocal e.g. regarding the link between ADC values and inflammation within CE lesions on T1 images [3–5].

Texture analysis (TA) has been investigated as an alternative quantitative approach to detect contrast-enhanced MS lesions [6], differentiate MS lesions from cerebral microangiopathies [7], characterize different sub-areas (core, rim) within lesions undergoing ‘active’ demyelination [8], differentiate between relapsing and remitting MS lesions [9], study perfusion characteristics of MS lesions [10], act as surrogate markers of lesion load and tissue integrity in MS [11,12], differentiate between primary progressive and relapsing-remitting MS phenotypes [13], differentiate MS lesions in patients with advanced *vs* mild disability status [14], make an outcome prognosis in patients with a clinically isolated syndrome [15], and assess the persistence or recovery of acute lesions in relapsing-remitting patients [16].

Texture refers to the spatial arrangement of primitive attributes, either visual or actual, of a surface. A brick in a brick wall,—or on a smaller scale the grains of a brick, constitutes a trivial example of a primitive attribute of the surface following a regular spatial arrangement. In medical imaging, primitive attributes are defined by image pixels, and texture refers to the visual appearance–or perceived properties–of the image, which can be more or less coarse, fine, uniform, granular, periodic or irregular. In mathematical terms, texture refers to the spatial distribution of the gray levels in the image matrix. Contrary to bricks in a brick wall, gray levels in medical images often follow more complex patterns, requiring high-order statistics or frequency approaches to characterize their arrangement. Numerical expressions have thus far been developed to assess the contrast, homogeneity, coarseness, and more broadly, all complex (non-visible to the human eye) variations in the distribution of the gray levels. All these numerical expressions have been referred to as ‘texture parameters’ [17,18].

In practice, TA generates a set of parameters that captures the pictorial content of the image, which may be useful for detection or classification purposes. The rationale behind the concept is that texture results from the process that created the surface. In MR imaging of MS lesions, it is assumed that the distribution of gray levels within the lesion results from the underlying ultra-structural properties of tissues affected by the disease processes, with or without therapeutic interventions [19]; a concept which has recently been validated by the histopathological analysis of brain white matter lesions appearing hyper-intense on T2-W MR images [20].

In a pioneering study, Yu et al. differentiated between enhanced and unenhanced brain MS lesions using a combination of 8 texture parameters with a sensitivity (Se) of 88% and a specificity (Sp) of 96% [21]. T2-W MR images were acquired from a spin-echo sequence on a 0.28T MR system in a small group of 8 patients. To our knowledge, this study has remained the only one specifically investigating TA as a potential alternative to CE T1-W imaging to identify acute inflammation within MS lesions.

One major reason to repeat this study design was that TA critically relies on image quality as well as on numerical solutions to measure it [22,23]. The experiment initially designed by Yu was repeated on a 3.0T system offering increased signal-to-noise ratio resulting in improved spatial resolution. Two TA methods and three statistical classifiers were implemented. A three-step assessment was undertaken: (i) texture and ADC parameters were compared in MS lesions *vs* normal appearing white matter (NAWM), (ii) the performance of individual parameters in identifying CE lesions was evaluated, and (iii), parameters were combined into multi-parametric models, the performances of which were assessed after cross-validation.

The availability of such an alternative model to contrast-enhanced MR imaging for monitoring inflammatory activity in MS patients is clearly beneficial in an era of economic constraints and for limiting systemic risks in persons with impaired kidney function.

## Materials and Methods

### Institutional EC board approval

The study was approved by our institutional ethics committee (CEBHF, Commission d’Ethique Biomédicale Hospitalo-Facultaire, Université Catholique de Louvain). Written informed consent of patients in the retrospective group (group 1) of the study was obtained for retrospectively reprocessing their imaging data extracted from the institutional PACS. Written consent was also obtained from patients in the prospective group (group 2) for repeating twice the T2-W acquisition before and after CA perfusion.

### Inclusion criteria, patients’ groups, and study design

Inclusion criteria in the study were as follows for the two (see below) patients’ groups: (i) a definite diagnosis of MS according to the 2010 revised McDonald's criteria for dissemination in space (DIS) and dissemination in time (DIT), (ii) a relapsing-remitting disease course, (iii) the presence of enhanced inflammatory lesions on CE-T1 images at the time of inclusion, and (iiii) the absence of any other co-existent neurological disorder. The study included two distinct groups of patients:

Patients from the retrospective group 1 were from the routine clinical practice in which patients receive intravenous injections of CA at a standard dose of 0.1 mmol.kg^-1^ of gadobenate dimeglumine (Multihance®, Bracco Imaging Europe®, Wavre, Belgium) outside the MR system. The timing of CA administration is synchronized with the end of the examination of the preceding patient. The MS patient is then introduced into the MR system almost immediately after CA perfusion. A standardized protocol is then applied with T2-W, FSE-FLAIR, and DWI sequences being acquired before the acquisition of CE T1-W images. Therefore, a constant delay ranging from 10 to 15 minutes between CA perfusion and T1 images acquisition is obtained. Twenty-one patients were extracted from the clinical database and PACS. 44 contrast-enhanced lesions, 37 unenhanced lesions and 44 regions of interest (ROI) in NAWM were delineated on CE T1-W images of patients in group 1.

Patients from the prospective group 2 had a different examination protocol. An intravenous access line was installed before examination. An initial pre-contrast T2-W sequence was acquired before CA perfusion. After CA perfusion of 0.1 mmol.kg^-1^ of gadobenate dimeglumine followed by 30 mL saline flush at a rate of 2 mL.s^-1^ with an automated power injector, a two-minute pause was observed and a similar protocol as for group 1 was thereafter applied including a repeated post-contrast T2-W sequence at the start. Both groups of patients had thus far post-contrast T1-W images in a similar delay ranging from 10 to 15 minutes after CA perfusion. Nine patients were recruited in this group, in which 14 contrast-enhanced lesions were delineated on CE T1-W images. TA was then performed on both pre- and post-contrast T2-W data.

The rationale for recruiting the two different groups of patients was the concern that TA was performed on post-contrast T2-W data in the main retrospective group 1. Since T2-W images were unaffected by CA perfusion at visual examination, the *a priori* hypothesis was that texture parameter values should also be unaffected by CA perfusion. To verify the hypothesis, the second prospective validation group 2 was subsequently recruited.

### MRI examinations

All MR examinations were performed using the same 3.0T whole body system (Achieva 3T, Philips Healthcare, Best, The Netherlands) with a 32-channel SENSE receiver head coil. Patients were imaged in the supine position using the following sequences: FSE T2-W sequence (scan parameters: TR/TE = 2565/80 ms, FOV = 230x184 mm, acquisition matrix = 384x246, slice thickness = 3 mm, interslice gap = 0 mm, 46 slices, in-plane spatial resolution after reconstruction = 0.45x0.45 mm), FSE FLAIR (scan parameters: TR/TE/TI = 11000/125/2800 ms, FOV = 230x183 mm, acquisition matrix = 352x189, slice thickness = 3 mm, interslice gap = 0 mm, 46 slices, in-plane spatial resolution after reconstruction = 0.51x0.51 mm), EPI DWI sequence (scan parameters: TR/TE = 4144/55 ms, pulse width = 12.1 ms, time between gradients = 26.3 ms, b = 0/100 s.mm^-2^, FOV = 230x230 mm, acquisition matrix = 128x101, slice thickness = 3 mm, interslice gap = 0 mm, 46 slices, in-plane spatial resolution after reconstruction = 0.90x0.90 mm) and 3D GRE T1-W sequence (scan parameters: TR/TE = 318/2.67 ms, FOV = 230x230 mm, acquisition matrix = 384x246, slice thickness = 3 mm, interslice gap = 0 mm, 46 slices, in-plane spatial resolution after reconstruction = 0.45x0.45 mm).

### Images analysis

**Fig 1:**
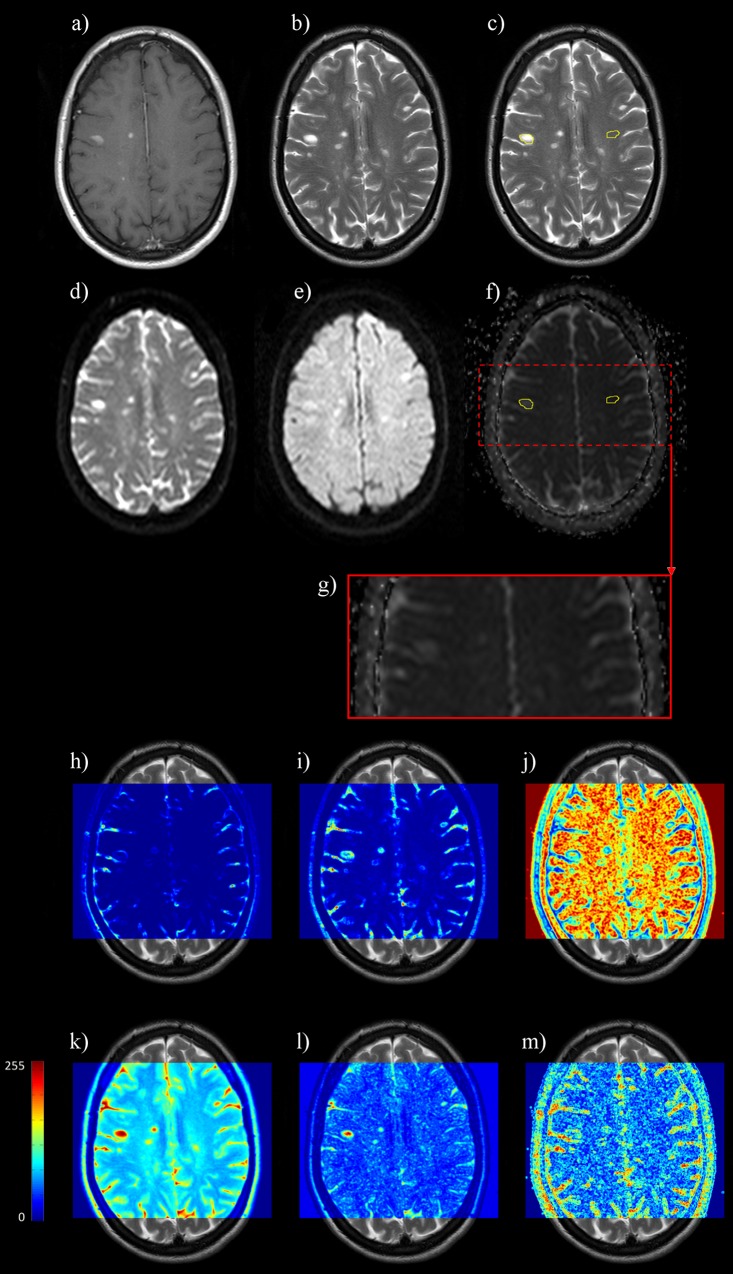
Method of ROI delineation and pixel-wise texture analysis from the gray level co-occurrence matrix (GLCM). a) Axial-transverse post-contrast T1-W image showing multiple enhanced lesions. b) T2-W image in similar slice location revealing additional hyper-intense unenhanced lesions. c) Segmentation on the same image as in b) of the largest active lesion as well as the contralateral mirrored area within NAWM. d) Corresponding DWI with gradient factor b_o_ = 0 s.mm^-2^. e) Corresponding DWI with gradient factor b = 1000 s.mm^-2^. f) ADC parametric map registered on anatomical T2-W image with superimposition of the ROIs drawn on c. g) Zoom of ADC mapped image on largest enhanced lesion (after erasing ROIs’ contours). h-m) Parametrical maps of the following texture parameter: h) contrast, i) correlation, j) homogeneity, k) sum average, l) sum variance and m) difference variance with mean value estimated on a 3x3 sliding window and normalized on the 0–255 range. Individual texture parameters revealed different local and regional statistical properties of the gray levels between MS lesions and NAWM and between enhanced and unenhanced MS lesions.

MR images of all patients (group 1 and 2) were consensually reviewed by both a senior resident and an experienced neuroradiologist (1 and 25 years of experience, respectively). MS lesions were categorized as enhanced or non-enhanced from the analysis of CE T1-W images. For each lesion, the slice with the largest cross-sectional dimensions was selected. The region of interest corresponding to the whole lesion was manually segmented on the T2-W image in a similar slice location (Fig 1). A contralateral mirrored ROI in NAWM was generated thereafter. Only lesions with homogeneous enhancement of 5 mm in diameter or more (according to the long axis) were considered for analysis.

Prior to the calculation of texture and ADC parameters, DWI was spatially registered with T2-W images using a rigid transformation [24], thereby replicating ROIs drawn on the T2-W images on the diffusion-weighted ones, which resulted in an anatomical match between texture and ADC ROIs.

The visual texture of ROIs was analyzed using the gray level co-occurrence matrix (GLCM) and the run length matrix (RLM) [17,25]. From the GLCM, nine texture parameters describing the gray levels’ interdependence in the image matrix were estimated. Computation parameters were: distance of one pixel between two neighboring pixels, average of the angular relationships on the four main directions, and five bits of gray levels. From the RLM, eleven texture parameters describing the distribution of runs of gray levels in the image were estimated with the same computation parameters. The mean value (over all pixels in the ROI) of the texture parameters was calculated. The list of parameters is given in Table 1.

**Table 1:** List of parameters used for the characterization of MS lesions.

PARAMETER TYPE		PARAMETER DESCRIPTION
**Diffusion**		
1	ADC	Apparent Diffusion Coefficient
**Texture**		
2*	Energy	Measure of local uniformity of gray levels
3*	Entropy	Measure of randomness of gray levels
4*	Contrast	Measure of the amount of gray levels variations
5*	Homogeneity	Measure of local homogeneity. It increases with less contrast
6*	Correlation	Measure of linear dependency of gray levels of neighboring pixels
7*	Inverse difference moment	Measure of local homogeneity of the gray levels
8*	Sum average	Measure of overall image brightness
9*	Sum variance	Measure of how spread out the sum of the gray levels of voxel pair is
10*	Difference in variance	Measure of variation in the difference in gray levels between voxel pairs
11†	SRE	Short Run Emphasis (first property of run-length distribution)
12†	LRE	Long Run Emphasis
13†	GLN	Gray-Level Nonuniformity
14†	RLN	Run-Length Nonuniformity
15†	RP	Run percentage
16†	LGRE	Low Gray-Level Run Emphasis
17†	HGRE	High Gray-Level Run Emphasis
18†	SRLGE	Short Run Low Gray-Level Emphasis
19†	SRHGE	Short Run High Gray-Level Emphasis
20†	LRLGE	Long Run Low Gray-Level Emphasis
21†	LRHGE	Long Run High Gray-Level Emphasis

*Parameters derived from the co-occurrence matrix

† Parameters derived from the run length matrix.

### Statistical analysis

Parameters values were expressed as the mean ± standard deviation. In the first analysis, texture parameters and ADC values within both enhanced and unenhanced MS lesions *vs* mirrored ROIs in NAWM were compared. A Wilcoxon signed rank test was performed as a non-parametric test because the normality of data distribution was not verified by the D’Agostino-Pearson test. A Bonferroni-type correction for performing three comparisons was applied and a *p*-value < 0.0167 was therefore considered as statistically significant.

In the second analysis, the performance of individual parameters in the discrimination of enhanced *vs* unenhanced lesions was assessed by a non-parametric receiver operating characteristic (ROC) curves analysis. Performance was interpreted as follows: AUC < 0.7 = poor, 0.7 ≤ AUC < 0.8 = fair, 0.8 ≤ AUC < 0.9 = good, 0.9 ≤ AUC < 1.0 = excellent. Parameters were ranked according to their performance by comparing Areas Under the ROC Curves (AUC).

In the third analysis, texture parameters and ADC were combined. Three multi-parametric classifiers were tested: linear discriminant analysis (LDA) [26], logistic regression models (LR) [27], and partial least squares (PLS) models [28]. As one cannot know *a priori* how many and which parameters played a significant role in the classification of MS lesions, all possible combinations of 2 to 21 parameters of the 21 parameters (20 texture parameters plus 1 ADC parameter constituting the independent variables of the analysis) were successively submitted to the classifiers. No variable reduction technique was used.

To estimate how accurately the classifiers would perform in practice, a leave-one-out cross-validation was applied [29]. The percentage of correctly classified enhanced lesions defined the classifier sensitivity (Se) and the percentage of correctly classified unenhanced lesions defined the classifier specificity (Sp). Se and Sp were used to identify the set of parameters yielding the best classification models of enhanced lesions.

All calculations were carried out with Matlab (Matlab R2011b, MathWorks®, Natick, MA, USA) and R-Project for Statistical Computing (http://www.r-project.org/). Open source codes “KeyRes-Technologies” and “grayrlmatrix” under Matlab were used for computing texture parameters. The software ImageJ (http://rsbweb.nih.gov/ij/) was used for the segmentation of the ROIs.

## Results

### Texture within NAWM vs MS lesions

Texture parameters and ADC values are given in Table 2 together with the significance levels for the statistical differences. Differences between enhanced lesions and NAWM, similarly to those between unenhanced lesions and NAWM, were statistically significant (*p* < 0.0167) for all parameters except for texture parameter LRHGE.

**Table 2:** Mean values (± standard deviation) of texture parameters and ADC parameter. The highly significant *p*-values observed demonstrate that the texture within NAWM is different when compared to MS lesions (enhanced or unenhanced), suggesting differences in the actual structure of the two tissues. Entropy was found higher in enhanced lesions when compared to unenhanced ones, suggesting that the randomness of gray levels was higher. This was confirmed by the lower Homogeneity and Energy in this type of lesion. Overall, this may suggest that the histologic substrate of enhancing lesions is more heterogeneous; an assumption that, however, needs to be investigated on experimental models allowing comparison between texture patterns and anatomopathological substrate to be confirmed.

	enhancing lesion ^(EL)^	non-enhancing lesion ^(NEL)^	NAWM	*p* ^EL *vs* NAWM^	*p* ^NEL *vs* NAWM^
**Energy**	24.3 ± 20.9	41.0 ± 19.5	84.3 ± 34.3	4.3 10^−13^	8.0 10^−9^
**Entropy**	208 ± 29.2	182 ± 26.1	131 ± 32.6	3.6 10^−13^	2.6 10^−9^
**Contrast**	11 ± 8.0	4.7 ± 3.4	1.6 ± 0.7	8.2 10^−15^	1.5 10^−11^
**Homogeneity**	121 ± 29.1	151 ± 22.1	189 ± 19.5	1.0 10^−13^	7.5 10^−10^
**Correlation**	52.8 ± 28.1	26.9 ± 13.9	7.3 ± 2.8	1.1 10^−15^	3.6 10^−14^
**Inv. Diff. Moment**	123 ± 32.5	156 ± 23.3	195 ± 18.7	1.1 10^−13^	5.9 10^−10^
**Sum average**	150 ± 32.9	122 ± 20.8	79.0 ± 12.6	1.1 10^−13^	5.7 10^−13^
**Sum variance**	81.7 ± 31.3	67.1 ± 21.7	45 ± 9.7	2.6 10^−10^	3.5 10^−7^
**Difference variance**	99.3 ± 29.8	77.3 ± 17.6	59.9 ± 10.3	7.2 10^−11^	4.4 10^−6^
**ADC (10** ^**−6**^ **mm** ^**2**^ **.s** ^**-1**^ **)**	1014 ± 227.8	1046 ± 168.4	751 ± 70.9	2.7 10^−10^	4.9 10^−12^
**SRE**	0.004 ± 0.002	0.006 ± 0.002	0.012 ± 0.003	8.6 10^−16^	6.9 10^−14^
**LRE**	322 ± 127	213 ± 74.1	96.0 ± 24.8	5.8 10^−16^	3.5 10^−14^
**GLN**	49 ± 44	86 ± 73	19 ± 22	2.0 10^−8^	2.8 10^−12^
**RLN**	13 ± 12	26 ± 14	20 ± 23	3.9 10^−3^	2.0 10^−3^
**RP**	0.75 ± 0.12	0.62 ± 0.10	0.50 ± 0.12	2.2 10^−12^	2.7 10^−6^
**LGRE**	0.80 ± 0.09	0.72 ± 0.08	0.56 ± 0.12	2.4 10^−13^	6.9 10^−9^
**HGRE**	2.73 ± 1.59	3.98 ± 1.58	7.94 ± 4.72	2.9 10^−12^	3.2 10^−6^
**SRLGE**	0.003 ± 0.001	0.004 ± 0.001	0.006 ± 0.002	6.4 10^−12^	4.2 10^−7^
**SRHGE**	0.01 ± 0.01	0.02 ± 0.01	0.09 ± 0.06	9.2 10^−16^	1.7 10^−12^
**LRLGE**	256 ± 116	152 ± 63	55 ± 21	3.2 10^−16^	5.6 10^−14^
**LRHGE**	851 ± 402	869 ± 371	734 ± 459	7.0 10^−2^	3.0 10^−2^

Statistical differences assessed with the Wilcoxon signed-rank test (significance level *p* < 0.0167).

### Performance of individual texture parameters

**Fig 2:**
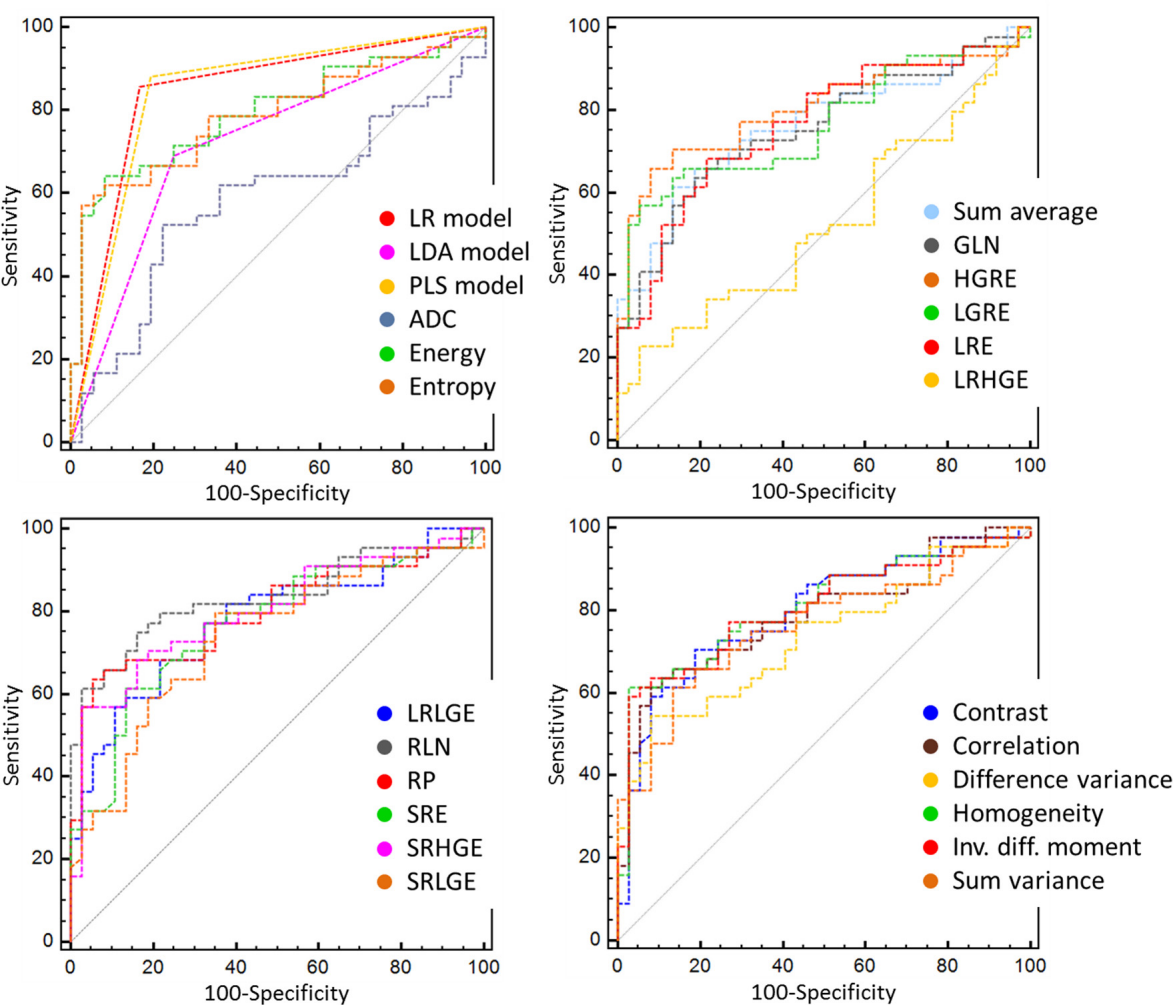
Receiver-Operating Characteristic analysis for evaluating the performance of individual parameters and multiparametric models in discriminating enhanced lesions from unhencanced lesions.

AUC values, sensitivity and specificity of selected cut-offs are given in Table 3, while ROC curves are displayed on Fig 2. ROC analysis showed that the performance of texture parameters ranged from poor (AUC ^sum variance^ = 0.638) to good (AUC ^RLN^ = 0.835). Individually, three parameters (Sum variance, LRHGE, ADC) did not perform better than a random classifier (*p* ^(AUC > 0.5)^ > 0.0167). A comparison of AUCs for parameters with a performance rated at least ‘good’ did not yield any statistically significant difference (*p* > 0.384, regardless of the comparison). A clear-cut ranking of these parameters according to their performance was therefore impossible, as was subsequently the identification of the best performing parameter.

**Table 3:** Performance of individual parameters in differentiating between enhanced and unenhanced MS lesions assessed by non-parametric receiver operating characteristic (ROC) curves. The significant *p*-values observed show that individual texture parameters are able to differentiate between the two types of MS lesions. Eight texture parameters displayed a level of individual performance that was at least ‘good’. None of these eight parameters was found to be significantly better performing than the other.

	AUC	Se (%)	Sp (%)	Cut-off	*p*-value ^1^
**Energy** *	0.805	65.9	91.9	22.5	<0.0001
**Entropy** *	0.800	59.1	97.3	211	<0.0001
**Contrast**	0.798	70.5	81.1	5.28	<0.0001
**Homogeneity** *	0.809	61.4	97.3	119	<0.0001
**Correlation**	0.789	61.4	91.9	44.3	<0.0001
**Inv. Diff. Moment** *	0.806	59.1	97.3	122	<0.0001
**Sum average**	0.763	61.4	86.5	143	<0.0001
**Sum variance**	0.638	63.6	63.2	67.6	0.0264
**Difference variance**	0.736	54.5	91.9	98.2	<0.0001
**ADC**	0.583	51.2	78.4	937	0.2061
**SRE**	0.770	61.4	86.5	0.0038	<0.0001
**LRE**	0.761	68.2	78.4	259	<0.0001
**GLN**	0.754	63.6	81.1	40.9	<0.0001
**RLN** *	0.835	75.0	83.8	14.2	<0.0001
**RP** *	0.800	63.6	94.6	0.723	<0.0001
**LGRE**	0.764	56.8	94.6	0.80	<0.0001
**HGRE** *	0.805	65.9	91.9	2.63	<0.0001
**SRLGE**	0.738	79.5	64.9	0.004	<0.0001
**SRHGE** *	0.800	56.8	97.3	0.008	<0.0001
**LRLGE**	0.778	68.2	78.4	189	<0.0001
**LRHGE**	0.526	22.7	94.6	492	0.6845

^1^ Parameters performing significantly better than a random classifier (*p* ^(AUC > 0.5)^ < 0.0167).

* Parameters with AUC ≥ 0.8 considered for a pair-wise comparison of performance.

### Performance of multi-parametric models

In the retrospective patient’s group (group 1), the best model from LDA classified enhanced lesions correctly in 31/44 cases (Se = 70%) and unenhanced lesions in 28/37 cases (Sp = 76%), relying on eight texture parameters (Entropy, Correlation, Sum Variance, SRE, LRE, RLN, RP, SRHGE) (Fig 2). The best model from PLS classified enhanced lesions correctly in 39/44 cases (Se = 88%) and unenhanced lesions in 30/37 cases (Sp = 81%), relying on two different sets of six texture parameters as follows: either the combination of (Correlation, Inverse Difference Moment, Sum Variance, GLN, RLN, LRHGE) or the combination of (Energy, Contrast, Correlation, Inverse difference Moment, GLN, LRHGE). According to the Youden index, the best model was based on LR and relied on ten texture parameters (Entropy, Homogeneity, Inverse Difference Moment, Difference Variance, LRE, RLN, RP, LGRE, SRHGE, LRLGE) through which enhanced lesions were classified correctly in 38/44 cases (Se = 86%) and unenhanced lesions were classified correctly in 31/37 cases (Sp = 84%). The best performing logistic regression model can be written as F(z) = e^z^/(1+e^z^), where F(z) is the probability of presence of the characteristic of interest and z is defined as follows:

z=−32.61−0.686*Entropy−8.365*Homogeneity+13.70*Inverse Difference Moment+2.144*Difference Variance−12.44*LRE+0.998*RLN−5.050*RP+4.974*LGRE−0.750*SRHGE+12.37*LRLGE

It should be noted that LDA, LR or PLS relying on other combinations and/or a larger number of parameters did not improve the classification.

Finally, the LR model previously identified as the best classification model was used in the prospective patients’ group (group 2) of the study. Enhanced lesions were correctly classified as active lesions in 14/14 (Se = 100%) either they were characterized with pre-contrast texture parameters or with post-contrast texture parameters, thereby demonstrating that, (i) CA perfusion has no substantial effect on texture parameters computed from T2-W images, and (ii) the 10 texture parameter model enabled identification of enhanced lesions with a high sensitivity.

## Discussion

The first observation drawn from the study was that all but one of the texture parameters were significantly different within white matter (WM) lesions than within normal appearing white matter (NAWM) as seen by visual examination of the T2-W images. This observation confirmed the ability of the technique to discriminate between normal and diseased WM and was consistent with previously published results in the field [20,30–32]. It also supported the assumptions that, (i) texture parameters are suitable for brain tissue classification and that, (ii) texture parameters can be used to evaluate local changes in the MR appearance of the white matter e.g. for monitoring the disease processes.

The second observation from the study was that the performance of eight of the individual texture parameters was evaluated as ‘good’ for differentiating lesions from NAWM. However, these mono-parametric models displayed high specificity but only fair sensitivity, thereby precluding accurate identification of acute inflammatory enhanced MS lesions. In turn, multi-parametric models based on texture parameters from T2-W MR images enabled differentiation between enhanced and unenhanced lesions with high sensitivity. We therefore confirmed the results reported by Yu et al [21] by using an updated MR technique, a larger data set data and by testing different statistical classifiers for the decision rule. The performance level of our analysis appeared lower (Se = 86% / Sp = 84% based on LR) compared to that reported in Yu’s study (Se = 88% / Sp = 96%). We assumed that differences in MR protocols (3.0T vs 0.28T, in-plane spatial resolution 0.45x0.45 mm vs 1x1 mm, slice thickness 3 mm vs 6 mm, higher homogeneity of the RF field with the Achieva system, higher signal to noise ratio with the 32-channel receiver-only SENSE head coil) yielded improved image quality of the T2-W images [33], which in turns affected texture parameter values and TA performance. The second reason for the difference in performance may arise from a difference in sample size and the absence of cross-validation in Yu’s study, though such validation is mandatory to obtain an unbiased estimate of the predictive accuracy. The use of techniques such as cross-validation, bootstrapping or Bayesian confidence interval should be systematic in such studies to obtain a reliable assessment of the classifier’s performance, which is both useful to estimate the relevance of the working hypothesis, and mandatory for clinical implementation.

The ADC parameter was not demonstrated to contribute significantly to the identification of inflammatory lesions as defined by enhancement on post-contrast T1-W images. Microstructural tissue damage in MS leads to an overall reduction of biological barriers of highly anisotropic healthy brain tissue. Disorganization and barrier breakdown (e.g. myelin) theoretically leads to an increase in free water diffusivity within damaged tissue compared to contralateral NAWM in patients or normal WM of healthy subjects. Several studies have confirmed an increase in water diffusivity within MS lesions [3,34–36], resulting from an increase in ‘free’ extracellular space either by extracellular edema at the acute inflammatory phase, or by demyelination at the chronic phase. However, the assumption that acute inflammation within enhanced lesions could display significantly different ADC values (or mean diffusivity values when diffusion tensor imaging is used) than within the chronic gliotic/demyelinated scar tissue of unenhanced lesions is still unverified and the reasons for variability in ADC value changes within enhanced lesions remain controversial [37,38]. Complex and mixed transient pathophysiological mechanisms such as acute inflammation, ongoing demyelination and maybe secondary remyelination may compete and modify changes in diffusivity in one direction or another.

There are methodological limitations to our study. This study is mainly retrospective using clinical material issued from routine practice. Sensitivity of TA was assessed on a limited number of lesions. Therefore, while our first set of data served for model learning, a larger set of patients’ data would be required to validate the performance of the model, and to confirm that CA administration had no substantial impact on texture parameters values in an additional group of patients accepting the repeated pre- and post-contrast T2-W acquisitions in the same imaging session.

Further tests in machine learning should be carried out since other types of classifiers than those tested in this study can be implemented, with a potential impact on the structure and performance of the model [39]. Several methods of texture analysis (2D or 3D) exist from which numerous texture parameters can be derived [8,11,17,26,40,41]. None of these approaches is superior to the other since their effectiveness basically relies on the visual properties of the images to which they are applied and on the task performed. Combining various texture parameters may improve the characterization of MS lesions as demonstrated by our data. However, increasing the number of parameters involves the use of variable reduction techniques prior to classification and the use of sophisticated machine learning classifiers, as well as larger training datasets. All these requirements may delay the routine clinical applicability of the processing.

Finally, although the ADC parameter was not useful in identifying enhanced lesions, other diffusion measurements such as fractional anisotropy, which has been reported to be significantly lowered in active lesions [42], could demonstrate relevance here.

In conclusion, this study provides additional evidence that texture analysis of T2-W MR images may be relevant in the identification of brain inflammatory activity in MS patients. These results are promising enough to trigger further investigation. Additional recruitment and tests are being performed to validate the structure and performance of the model. Such a fully automated post-processing method implemented using a computer-aided diagnosis (CAD) system for clinical use could be used for the innocuous and non-invasive detection of subtle changes in texture properties within white matter during relapses and the monitoring of the overall MS disease process.
